# Computational pipeline to identify and characterize functional mutations in ornithine transcarbamylase deficiency

**DOI:** 10.1007/s13205-014-0216-y

**Published:** 2014-04-24

**Authors:** R. Magesh, C. George Priya Doss

**Affiliations:** 1 Department of Biotechnology, Faculty of Biomedical Sciences, Technology and Research, Sri Ramachandra University, Chennai, 600116 India; 2 Medical Biotechnology Division, School of Biosciences and Technology, VIT University, Vellore, India

**Keywords:** *OTC*, OTCD, SIFT, PolyPhen 2, I-Mutant 3, SNPs&Go, PhD-SNP

## Abstract

Ornithine transcarbamylase (OTC) (E.C. 2.1.3.3) is one of the enzymes in the urea cycle, which involves in a sequence of reactions in the liver cells. During protein assimilation in our body surplus nitrogen is made, this open nitrogen is altered into urea and expelled out of the body by kidneys, in this cycle *OTC* helps in the conversion of free toxic nitrogen into urea. Ornithine transcarbamylase deficiency (OTCD: OMIM#311250) is triggered by mutation in this *OTC* gene. To date more than 200 mutations have been noted. Mutation in *OTC* gene indicates alteration in enzyme production, which upsets the ability to carry out the chemical reaction. The computational analysis was initiated to identify the deleterious nsSNPs in *OTC* gene in causing OTCD using five different computational tools such as SIFT, PolyPhen 2, I-Mutant 3, SNPs&Go, and PhD-SNP. Studies on the molecular basis of *OTC* gene and OTCD have been done partially till date. Hence, in silico categorization of functional SNPs in *OTC* gene can provide valuable insight in near future in the diagnosis and treatment of OTCD.

## Introduction

Ornithine transcarbamylase (OTC) catalyzes the formation of citrulline from carbamoyl phosphate and l-ornithine in the urea cycle, deleterious mutations in the human *OTC* gene disrupts the formation and produces clinical hyperammonemia, which can also lead to encephalopathy with subsequent neurological symptoms or even death. Ornithine transcarbamylase deficiency (OTCD) is the most common inborn error of urea cycle showing X-linked inheritance, which occurs at a predictable frequency of 1 in 14,000 births. Affected individuals show elevated levels of ammonia in their plasma and amplified urinary flow of orotic acid (Lopes-Marques et al. 2012). Males with OTCD show neonatal ammonia intoxication with severe or fatal neurological damage. Those with limited enzymatic OTCD may perhaps have a normal life span, but are at the peak intended for stress-induced hyperammonemic emergencies and incremental neurological damage. Females are carriers who might be asymptomatic, but often show some amount of protein intolerance (Maddalena et al. 1988). The human *OTC* gene is found on the short arm of the X chromosome with its cytogenetic location being Xp21.1. The size of the gene is 73 kb with an open reading frame of 1,062 nucleotides and holds 10 exons interjected by 9 introns of highly variable size. The *OTC* gene is expressed entirely in the liver and small intestinal mucosa. It translates a precursor OTC protein containing 354 amino acids and the amino end contains a spearhead sequence of 32 amino acids, which is cleaved in two steps upon integration into the mitochondrial matrix (Ogino et al. 2007). A polymorphism is a germline variation in the nucleotide base of the DNA molecule. As a rule of thumb inheritable variation is termed, a polymorphism if it is present at an allele frequency greater than 1 % in the general population, otherwise, at lower frequencies, it is considered as germline mutation (Strachan and Read 1996). Genetic polymorphisms are present throughout the genome of human. The most common type of polymorphism is single nucleotide polymorphism (SNP) that can occur in the frequency of about 1 out of every 300 nucleotide base pairs, and there are probably more than 10 million SNPs in the human population (The international HapMap and Consortium 2006). Polymorphisms can occur in both coding and non-coding region of the genes and may sometimes, particularly those within exons, have an impact on the structure and function of the protein coded by a particular gene, especially in those cases when the polymorphism leads to an amino acid substitution in evolutionarily conserved functional region of the protein.

A polymorphism that takes to an amino acid substitution and is present within an active site of an enzyme, at a substrate-binding site, a DNA-binding site or in other areas of the protein domains may affect the function of the encoded protein. This is particularly correct if the substituted amino acid has a different 3D structure or electrical charge than the wild-type amino acid, as this will alter the conformation or affinity of the enzyme, and make it non-functional, or more or less efficient than the wild-type protein (AliOsman et al. 1997; Hadi et al. 2000; Matullo et al. 2001; Pemble et al. 1994).

The loss of stability of proteins is one of the foremost causes of disease. As the proteins are only marginally stable, even small effects on stability alter the thermodynamic equilibrium to make the folded state unstable. Mutational data show that mutations often, if not in the majority of cases, cause significant changes to protein stability which are often on the order of magnitude of the absolute stability of the protein (Guerois et al. 2002). Lowered stability leads to a reduction in a protein’s effective concentration, which in turn causes deficiencies in its ability to perform its biochemical function (Pakula et al. 1986).

Mutations in this *OTC* gene are the main reason for OTCD. Deleterious non-synonymous single nucleotide polymorphism (nsSNP) analysis for the *OTC* gene has not been projected computationally until now, while they are the center for new investigators. Therefore, in this work, the computational methods namely SIFT, PolyPhen 2, I-Mutant 3, SNPs&Go, and PhD-SNP were used to identify the deleterious nsSNPs that are expected to be affecting the function and structure of the OTC protein.

## Materials and methods

### Dataset used for SNP annotation

Human *OTC* gene information data were collected from Online Mendelian Inheritance in Man (OMIM) (Amberger et al. 2009) and Entrez Gene on National Centre for Biological Information (NCBI). The SNP information of CBS was retrieved from the NCBI dbSNP (Sherry et al. 2001), and SWISS-Prot databases (Amos and Rolf 1996). Protein 3D structure was obtained from protein data bank (PDB) (Berman et al. 2000).

### Sorting intolerant from tolerant (SIFT)

Sequence homology-based tool SIFT predicts the functional importance of amino acid substitution based on the alignment of highly similar orthologous and/or paralogous protein sequences. SIFT scores were designated as intolerant (0.00–0.05), potentially intolerant (0.051–0.10), borderline (0.101–0.20), or tolerant (0.201–1.00) (Kumar et al. 2009).

### PolyPhen 2

PolyPhen2 (Polymorphism Phenotyping) predicts the functional effect of amino acid changes by considering evolutionary conservation, the physico-chemical differences, and the proximity of the substitution to predicted functional domains and/or structural features. A mutation is classified as “probably damaging” if the probabilistic score is above 0.85–1, mutation is classified as “possibly damaging” if the probabilistic score is above 0.15–0.84, and the remaining mutations are classified as benign (Adzhubei et al. 2010).

### I-Mutant 3

SVM-based method I-Mutant 3 predicts the protein stability changes upon a single point mutation. It provides free energy change (DDG), which is calculated from the unfolding Gibbs free energy change of the mutated protein minus the unfolding free energy value of the native protein (Kcal/mol). It classifies the predictions in three classes: If DDG is <−0.5 = large decrease of stability, If DDG is between −0.5 and 0.5 = neutral stability and If DDG is >0.5 = large increase of stability (Capriotti et al. 2005).

### SNPs&GO

It is a method based on SVMs that predict disease-associated mutations from protein sequence, evolutionary information and functions as encoded in the gene ontology terms. Moreover, it is a server for the predicting single point mutations, which cause disease in humans (Calabrese et al. 2009).

### PhD-SNP

PhD-SNP uses SVM-Sequence method and SVM profile to classify the mutation into disease related and neutral polymorphisms. It predicts if the given nsSNP has pathological effect based on the local sequence environment of the mutation. It uses the most accurate mode that enables both sequence and evolutionary profiles (Capriotti et al. 2006).

### Structural analysis

To evaluate the structural stability of native and mutant, protein structure analysis was performed. We used the web resource dbSNP to identify the protein coded by OTC. We also confirmed the mutation positions and the mutation residues from this server. These mutation residues and their corresponding positions were in complete agreement with the results obtained from the in silico prediction methods SIFT, PolyPhen 2, I-Mutant 3, SNPs&GO and PhD-SNP. The mutation was performed using SWISS-PDB viewer (Guex and Peitsch 1997), and energy minimization for 3D structures was performed by NOMAD-Ref server (Lindahl et al. 2006). This server uses Gromacs as default force field for energy minimization based on the methods of steepest descent, conjugate gradient and L-BFGS methods. Conjugate gradient method was used for optimizing the 3D structures. Deviation between the two structures was evaluated by their Root Mean Square Deviation (RMSD) values.

## Results

A total of about 195 SNPs were collected and their deleterious natures were analyzed by various computational methods.

### Analysis of deleterious SNPs using evolutionary-based prediction methods

SIFT algorithm calculates whether an amino acid replacement may have an impact on protein function by aligning similar proteins and calculating a score which tells the evolutionary conservation status of the amino acid of our interest. SIFT scores were obtained for 195 SNPs. SIFT scores were classified as intolerant (0.00–0.05), potentially intolerant (0.051–0.10), borderline (0.101–0.20), and tolerant (0.201–1.00). Approximately 115 (58.97 %) of the SNPs exhibit SIFT scores of 0.0. Another 45 (23 %) of the variants have scores between 0.01 and 0.05. Thus, 82 % of the SNPs are classified as ‘‘intolerant’’ by SIFT. The remaining SNPs were found to be “tolerant”. SIFT gave a prominent result with an 82 % of predictions to be deleterious.

### Analysis of deleterious SNPs using structure-based prediction methods

The influences of nsSNPs in protein function were tested using structure-based predictors by applying it to three different methods. The structural levels of changes of 195 nsSNPs were determined by PolyPhen 2. To provide an outline of the distribution of PolyPhen 2 scores, the scores are distributed into three groups. PolyPhen 2 scores falling between 0.85 and 1 are expected to be ‘‘probably damaging’’ to protein structure and function. 157 (80 %) of the nsSNPs were found to have scores in the above-mentioned category. An additional 19 (9.7 %) of the variants exhibited PolyPhen 2 scores of 0.2–0.84, indicative of variants that are ‘‘possibly damaging’’ to protein function, and the remaining 17 (8.7 %) nsSNPs that scored less than 0.02 were designated as ‘‘benign’’. SNPs&GO makes use of sequence and evolutionary information to predict whether a mutation is disease related or not by developing the protein functional annotation. The protein sequences with corresponding UniProt accession numbers were submitted along with their corresponding mutational position, wild-type and mutant-type residue as input to the server. 98 % of the nsSNPs were designated as “disease”. These mutants are found to be disease causing. PhD-SNP predicts the given nsSNPs have pathological effects based on the local sequence environment of the mutation. It classifies the SNPs into disease or neutral based on the most accurate mode that uses both sequence and evolutionary profiles. It showed 64 % of nsSNPs were likely to cause disease on mutation.

### Prediction of stability changes

Mutated proteins involved in diseases show a stability change. Predicting the protein stability upon mutation is necessary for understanding structure function relationship of protein. Generally, the stability of a protein is represented by the change in the Gibbs free energy upon folding (Δ*G*), where an increasingly negative number represents greater stability. Single amino acid substitution in a protein sequence can result in a significant change in the protein’s stability (ΔΔ*G*), where a positive ΔΔ*G* represents a destabilizing mutation and a negative value represents a stabilizing mutation. All the 195 nsSNPs submitted to pathogenic prediction tools were also subjected to protein stability analysis by I-Mutant 3.0. It gave an estimation of 107 nsSNPs (54 %) caused decreased stability, 48 SNPs (24 %) were neutral to the mutation, and 39 SNPs (20 %) increased the stability of protein after mutation. Out of 195 nsSNPs, 92 nsSNPs (47 %) were predicted to be positive by SIFT, PolyPhen 2, I-Mutant 3, SNPs&Go, and PhD-SNP (Table 1).

**Table 1:** List of nsSNPs in OTC found to be deleterious/neutral by computational methods

S. no.	rs IDs	Variants	SIFT	PolyPhen 2	I-Mutant 3	SNPs&GO	PhD-SNP
1	rs72552295	M1T	0.01	0.895	−0.28	Disease	Neutral
2	rs72552296	M1I	0.03	0.465	0.69	Disease	Neutral
3	rs67752076	M1V	0.14	0.064	0.61	Disease	Neutral
4	rs137853257	R10P	NA	NA	–1.12	NA	Neutral
5	rs148660170	R23Q	0.15	0.139	–0.97	Disease	Neutral
6	rs68031618	R26Q	0.59	0.002	–0.84	Disease	Neutral
7	rs199858968	G28E	0.06	0.999	–0.82	Disease	Neutral
8	rs72554306	G39C	0.01	1	–2.15	Disease	Neutral
9	rs72554307	R40C	0	1	1.16	Disease	Disease
10	rs72554308	R40H	0.03	0.54	–0.28	Disease	Neutral
11	rs74518351	D41G	0.31	0.103	–2.91	Disease	Neutral
12	**rs72554309**	**L43F**	**0.01**	**1**	**–1.08**	**Disease**	**Disease**
13	rs72554310	T44I	0.01	1	–2.64	Disease	Neutral
14	rs72554311	L45V	0.01	0.967	–0.7	Disease	Neutral
15	**rs72554312**	**L45P**	**0**	**1**	**–1.26**	**Disease**	**Disease**
16	rs1800321	K46R	0.49	0.187	–1.02	Disease	Disease
17	rs67939655	N47T	0.01	0.07	–0.66	Disease	Disease
18	**rs72554315**	**F48S**	**0.01**	**1**	**–0.12**	**Disease**	**Disease**
19	**rs72554316**	**T49P**	**0**	**0.922**	**–1.82**	**Disease**	**Disease**
20	rs201802621	G50A	1	0.02	–0.01	Disease	Neutral
21	**rs72554317**	**E52G**	**0**	**1**	**–2.37**	**Disease**	**Disease**
22	**rs72554318**	**E52D**	**0.01**	**1**	**–1.61**	**Disease**	**Disease**
23	**rs66521141**	**E52K**	**0**	**1**	**–1.69**	**Disease**	**Disease**
24	**rs66677059**	**I53T**	**0**	**1**	**–2.68**	**Disease**	**Disease**
25	rs72554319	Y55D	0.23	0.919	–0.42	Disease	Neutral
26	rs72554320	M56T	0	0.197	–0.34	Disease	Neutral
27	**rs72554321**	**L57Q**	**0**	**1**	**–0.42**	**Disease**	**Disease**
28	**rs72554323**	**S60L**	**0**	**1**	**–1.77**	**Disease**	**Disease**
29	**rs72554324**	**L63P**	**0.01**	**1**	**–0.99**	**Disease**	**Disease**
30	rs72554325	I67R	0.22	1	–1.61	Disease	Neutral
31	rs72554328	L76S	0.41	0.994	–0.08	Disease	Disease
32	rs72554329	L77F	0	0.998	0.05	Disease	Disease
33	**rs72554331**	**G79E**	**0**	**1**	**–1.51**	**Disease**	**Disease**
34	**rs72554332**	**K80E**	**0.03**	**0.468**	**–1.6**	**Disease**	**Disease**
35	rs72554333	K80 N	0	0.997	–2.25	Disease	Neutral
36	**rs72554336**	**G83R**	**0**	**0.923**	**–1.04**	**Disease**	**Disease**
37	**rs72554337**	**G83D**	**0**	**0.824**	**–1.85**	**Disease**	**Disease**
38	**rs72554338**	**E87K**	**0**	**0.96**	**–1.56**	**Disease**	**Disease**
39	rs72554339	K88N	0	1	–2.09	Disease	Neutral
40	**rs72554340**	**S90G**	**0**	**1**	**–1.65**	**Disease**	**Disease**
41	**rs72554341**	**S90N**	**0**	**1**	**–0.83**	**Disease**	**Disease**
42	rs72554342	S90R	0	1	–2.7	Disease	Neutral
43	rs67418243	R92G	0	0.979	–0.37	Disease	Neutral
44	**rs66550389**	**R92Q**	**0**	**1**	**–0.55**	**Disease**	**Disease**
45	**rs72554344**	**T93A**	**0**	**0.966**	**–1.39**	**Disease**	**Disease**
46	rs72554345	R94T	0	1	–0.6	Disease	Neutral
47	rs72554346	L95S	0	0.283	–2.74	Disease	Disease
48	**rs184053962**	**S96R**	**0**	**1**	**–0.86**	**Disease**	**Disease**
49	rs72554347	E98K	0	1	0.85	Disease	Neutral
50	rs72554349	G100D	0	1	0.19	Disease	Disease
51	rs1133135	F101L	0.3	0.208	0.61	Disease	Neutral
52	rs72554350	A102E	0.29	0.989	1.09	Disease	Neutral
53	rs72554351	G105V	0	1	0.14	Disease	Disease
54	**rs72554352**	**G106R**	**0**	**1**	**–2.08**	**Disease**	**Disease**
55	**rs67651903**	**G106E**	**0**	**1**	**–1.11**	**Disease**	**Disease**
56	**rs1800324**	**L111P**	**0**	**1**	**–0.47**	**Disease**	**Disease**
57	**rs66539573**	**H117R**	**0.02**	**0.993**	**–1.43**	**Disease**	**Disease**
58	rs72554356	T125M	0.21	0.688	–0.84	Disease	Neutral
59	**rs72554358**	**D126G**	**0**	**1**	**–0.68**	**Disease**	**Disease**
60	**rs140046498**	**R129C**	**0**	**0.9999**	**–1.87**	**Disease**	**Disease**
61	**rs66656800**	**R129H**	**0.02**	**1**	**–2.46**	**Disease**	**Disease**
62	**rs72556252**	**L131S**	**0**	**1**	**–2.09**	**Disease**	**Disease**
63	rs72556253	S132P	0	1	0.95	Disease	Disease
64	rs72556254	S132F	0	1	1.37	Disease	Disease
65	rs72556256	A135E	0.09	0.122	0.53	Disease	Disease
66	rs72556257	D136V	0	0.996	0.86	Disease	Disease
67	rs72556258	A137T	0.04	0.761	0.19	Disease	Neutral
68	rs72556259	L139S	0	1	–0.06	Disease	Neutral
69	**rs72556260**	**A140P**	**0.04**	**1**	**–0.46**	**Disease**	**Disease**
70	**rs68026851**	**R141Q**	**0**	**1**	**–0.27**	**Disease**	**Disease**
71	**rs67960011**	**R141G**	**0**	**1**	**–2.51**	**Disease**	**Disease**
72	**rs72556261**	**V142E**	**0**	**1**	**0.55**	**Disease**	**Disease**
73	rs67016166	L148S	0	1	0.1	Disease	Neutral
74	rs66741318	L148F	0	1	0.84	Disease	Disease
75	rs72556265	L151R	0	1	–0.21	Disease	Neutral
76	rs72556266	A152V	0.02	0.791	1.19	Disease	Neutral
77	rs72556268	A155E	0	0.999	–0.24	Disease	Neutral
78	**rs67890094**	**A155P**	**0.01**	**0.999**	**–1.22**	**Disease**	**Disease**
79	**rs72556269**	**I159T**	**0**	**0.99**	**–3.21**	**Disease**	**Disease**
80	**rs67954347**	**I160N**	**0**	**0.996**	**–0.25**	**Disease**	**Disease**
81	**rs72558497**	**N161K**	**0**	**1**	**–1.25**	**Disease**	**Disease**
82	rs72556270	N161D	0	1	0.44	Disease	Disease
83	rs72556271	N161S	0.03	0.998	0.17	Disease	Neutral
84	rs72556272	G162E	0	1	–1.58	Disease	Neutral
85	rs66626662	G162R	0	1	–0.57	Disease	Neutral
86	**rs72556273**	**S164P**	**0**	**1**	**–0.34**	**Disease**	**Disease**
87	rs72556275	D165Y	0	1	–0.73	Disease	Neutral
88	**rs72556276**	**H168Q**	**0**	**1**	**–1.47**	**Disease**	**Disease**
89	**rs72556277**	**P169A**	**0**	**1**	**–0.9**	**Disease**	**Disease**
90	**rs72556278**	**P169L**	**0**	**1**	**–0.6**	**Disease**	**Disease**
91	**rs72556279**	**I172F**	**0.01**	**1**	**–0.91**	**Disease**	**Disease**
92	rs72556280	I172M	0	0.964	–0.5	Disease	Neutral
93	**rs72556281**	**A174P**	**0**	**1**	**–1.52**	**Disease**	**Disease**
94	**rs68033093**	**D175G**	**0.01**	**1**	**–3.78**	**Disease**	**Disease**
95	**rs72556282**	**Y176H**	**0**	**0.999**	**–1.39**	**Disease**	**Disease**
96	**rs72556283**	**Y176C**	**0**	**1**	**–1.39**	**Disease**	**Disease**
97	rs148961194	L177F	0.02	1	–0.13	Disease	Neutral
98	**rs72556284**	**T178M**	**0**	**1**	**–0.51**	**Disease**	**Disease**
99	**rs72556286**	**L179P**	**0**	**1**	**–0.63**	**Disease**	**Disease**
100	**rs72556290**	**E181G**	**0**	**0.793**	**–0.62**	**Disease**	**Disease**
101	rs143746493	H182Q	0.14	1	–1.93	Disease	Neutral
102	rs72556291	H182L	0.27	1	–1.06	Disease	Neutral
103	**rs72556292**	**Y183D**	**0.04**	**1**	**–1.91**	**Disease**	**Disease**
104	**rs72556294**	**G188R**	**0**	**1**	**–2.08**	**Disease**	**Disease**
105	**rs72556295**	**G188V**	**0**	**1**	**–0.91**	**Disease**	**Disease**
106	rs72556296	L191F	0.13	0.202	–0.08	Disease	Disease
107	**rs72556297**	**L191R**	**0**	**0.998**	**–0.03**	**Disease**	**Disease**
108	**rs72556298**	**S192R**	**0**	**0.999**	**–0.57**	**Disease**	**Disease**
109	rs67284661	W193R	0	1	–0.91	Disease	Neutral
110	**rs67294955**	**G195R**	**0**	**1**	**–1.97**	**Disease**	**Disease**
111	rs72556300	D196V	0	1	1.37	Disease	Disease
112	**rs66642398**	**D196N**	**0**	**1**	**–0.09**	**Disease**	**Disease**
113	**rs72556301**	**G197R**	**0**	**1**	**–0.09**	**Disease**	**Disease**
114	rs72556302	G197E	0	1	0.26	Disease	Disease
115	rs72558403	N198I	0	1	0.26	Disease	Disease
116	**rs72558404**	**N198K**	**0**	**1**	**–0.84**	**Disease**	**Disease**
117	**rs72558405**	**N199D**	**0**	**1**	**–0.97**	**Disease**	**Disease**
118	**rs72558406**	**N199S**	**0**	**1**	**–0.91**	**Disease**	**Disease**
119	**rs72558407**	**L201P**	**0.01**	**1**	**–0.31**	**Disease**	**Disease**
120	rs72558408	H202Y	0.03	1	–2.74	Disease	Neutral
121	**rs72558409**	**H202P**	**0**	**0.998**	**–2.84**	**Disease**	**Disease**
122	**rs72558410**	**S203C**	**0**	**1**	**–3.27**	**Disease**	**Disease**
123	rs72558411	M205V	0.03	0.904	–0.64	Disease	Neutral
124	rs72558412	M206R	0.03	0.998	–2.5	Disease	Neutral
125	rs72558413	M206I	0.13	0.069	–0.95	Disease	Neutral
126	rs72558414	S207N	0.06	0.805	–0.31	Disease	Disease
127	rs72558415	S207R	0.01	1	0.42	Disease	Neutral
128	rs72558416	A208T	0.08	1	–0.05	Disease	Neutral
129	rs72558417	A209V	0.04	1	1.21	Disease	Neutral
130	rs72558418	K210Q	0.03	0.897	0.57	Disease	Neutral
131	rs72558419	M213L	0.17	0.721	–0.67	Disease	Neutral
132	rs72558420	H214Y	0.02	0.98	0.4	Disease	Neutral
133	rs72558421	L215F	0.14	0.994	–0.41	Disease	Disease
134	rs72558423	Q216E	0.97	0.663	–1.03	Disease	Disease
135	**rs72558424**	**A217E**	**0**	**0.994**	**–1.02**	**Disease**	**Disease**
136	**rs72558425**	**P220A**	**0**	**1**	**–1.05**	**Disease**	**Disease**
137	**rs72558426**	**P220L**	**0**	**1**	**–1.15**	**Disease**	**Disease**
138	rs72558428	P225T	0.27	0.954	–0.29	Disease	Neutral
139	rs67120076	P225R	0.03	0.975	0.12	Disease	Disease
140	rs72558429	A233V	0	0.998	–1.49	Disease	Neutral
141	rs4385598	Q235Q	0.83	NA	NA	NA	NA
142	rs67283833	E239G	0.16	0.85	–0.47	Disease	Disease
143	rs72558435	T242I	0.05	1	–0.23	Disease	Disease
144	**rs72558436**	**L244Q**	**0**	**0.854**	**–0.11**	**Disease**	**Disease**
145	**rs72558437**	**T247K**	**0.03**	**0.974**	**–0.55**	**Disease**	**Disease**
146	**rs67330615**	**A253T**	**0**	**1**	**–2.18**	**Disease**	**Disease**
147	rs72558440	H255P	0.02	0.017	–0.9	Disease	Disease
148	**rs142592280**	**N258S**	**0**	**0.688**	**–1.72**	**Disease**	**Disease**
149	rs72558441	L260S	0	1	0.24	Disease	Disease
150	**rs67333670**	**T262K**	**0**	**1**	**–0.62**	**Disease**	**Disease**
151	rs72558442	D263N	0	1	0.33	Disease	Neutral
152	**rs72558443**	**D263G**	**0**	**1**	**–1.23**	**Disease**	**Disease**
153	**rs72558444**	**T264A**	**0.01**	**0.998**	**–2.53**	**Disease**	**Disease**
154	**rs67156896**	**T264N**	**0.03**	**0.902**	**–1.76**	**Disease**	**Disease**
155	**rs72558445**	**W265R**	**0**	**0.996**	**–0.87**	**Disease**	**Disease**
156	**rs72558446**	**W265L**	**0**	**1**	**–0.26**	**Disease**	**Disease**
157	**rs72558448**	**S267R**	**0**	**1**	**–0.51**	**Disease**	**Disease**
158	**rs72558449**	**M268T**	**0**	**1**	**–1.98**	**Disease**	**Disease**
159	**rs72558450**	**G269E**	**0**	**1**	**–1.43**	**Disease**	**Disease**
160	**rs72558451**	**Q270E**	**0.01**	**0.923**	**–1.13**	**Disease**	**Disease**
161	rs1800328	Q270P	0	0.284	–2.14	Disease	Disease
162	**VAR_004927**	**Q270R**	**0**	**0.977**	**–1.14**	**Disease**	**Disease**
163	**rs72558454**	**R277W**	**0**	**1**	**–0.46**	**Disease**	**Disease**
164	**rs66724222**	**R277Q**	**0**	**1**	**–0.35**	**Disease**	**Disease**
165	rs72558461	W298S	0.05	0.987	–0.96	Disease	Neutral
166	rs72558462	L301F	0.01	0.997	0.16	Disease	Disease
167	rs72558463	H302Y	0	1	1.2	Disease	Disease
168	rs67993095	H302R	0	1	–0.1	Disease	Neutral
169	rs67870244	H302K	0	1	0.21	Disease	Neutral
170	rs72558464	C303Y	0	1	0.74	Disease	Disease
171	rs67468335	C303R	0	1	0.28	Disease	Disease
172	rs72558465	L304F	0	0.999	1.14	Disease	Disease
173	**rs67501347**	**P305H**	**0**	**1**	**–2.28**	**Disease**	**Disease**
174	**rs72558467**	**E310G**	**0**	**1**	**–3.46**	**Disease**	**Disease**
175	rs72558468	V311M	0	1	–0.27	Disease	Neutral
176	rs137899554	E314A	0.11	0.003	–1.44	Disease	Neutral
177	**rs72558470**	**V315F**	**0**	**0.999**	**–0.97**	**Disease**	**Disease**
178	**rs67414444**	**V315D**	**0**	**1**	**–3.9**	**Disease**	**Disease**
179	rs72558471	F316S	0	0.999	–0.05	Disease	Neutral
180	**rs72558472**	**S318F**	**0.04**	**0.555**	**–0.51**	**Disease**	**Disease**
181	**rs72558474**	**R320L**	**0.01**	**0.948**	**–1.64**	**Disease**	**Disease**
182	**rs72558476**	**E326K**	**0**	**1**	**–0.52**	**Disease**	**Disease**
183	rs72558478	R330G	0	1	–2.29	Disease	Neutral
184	**rs72558480**	**W332R**	**0.03**	**1**	**–0.84**	**Disease**	**Disease**
185	rs72558486	A336S	0.16	0.791	0.41	Disease	Disease
186	rs72558487	V337L	0.32	0.007	1.53	Disease	Neutral
187	rs199568993	M338L	0.56	0.012	1.21	Disease	Neutral
188	rs72558488	V339L	0.3	0.001	1.1	Disease	Neutral
189	rs72558489	S340P	0.09	0.996	0.77	Disease	Neutral
190	**rs72558490**	**L341P**	**0**	**1**	**–0.85**	**Disease**	**Disease**
191	rs72558491	T343K	0.92	0.101	–0.48	Disease	Neutral
192	rs72558492	Y345C	0.01	0.999	–1.92	Disease	Neutral
193	rs66469337	Y345H	0.23	0.042	–2.64	Disease	Neutral
194	rs72558493	P347T	0.02	0.997	–0.62	Disease	Neutral
195	rs72558495	F354C	0	0.791	–0.22	Disease	Neutral

rs IDs highlighted in bold were found to be deleterious by SIFT, PolyPhen 2, I-Mutant 3, SNPs&GO and PhD-SNP

### Structural analysis

According to the computational prediction in *OTC* gene, structural analysis was performed for the five highly deleterious variants by modeling mutant structures using native X-ray crystallographic structure (PDB ID: 1OTH). An energy minimization study gives the information about the protein structure stability. We checked the total energy for native- and mutant-type structures. In *OTC* gene, mutation occurred for the native protein in ‘A’ chain of protein structure at position D126G, R141Q, A174P, T178M and G195R. It can be seen that the total energy value and RMSD of native-type and mutant-modeled structures (D126G, A174P, and G195R) were found to be higher (Table 2).

**Table 2:** Summary of deleterious nsSNPs in the coding region of *OTC* gene

RS IDS	Amino acid position	SIFT	PolyPhen 2	I-Mutant 3	SNPs&GO	PHD-SNP
rs72554358	D126G	0	1	–0.68	Disease	Disease
rs68026851	R141Q	0	1	–0.27	Disease	Disease
rs72556281	A174P	0	1	–1.52	Disease	Disease
rs72556284	T178M	0	1	–0.51	Disease	Disease
rs67294955	G195R	0	1	–1.97	Disease	Disease

**Fig. 1:**
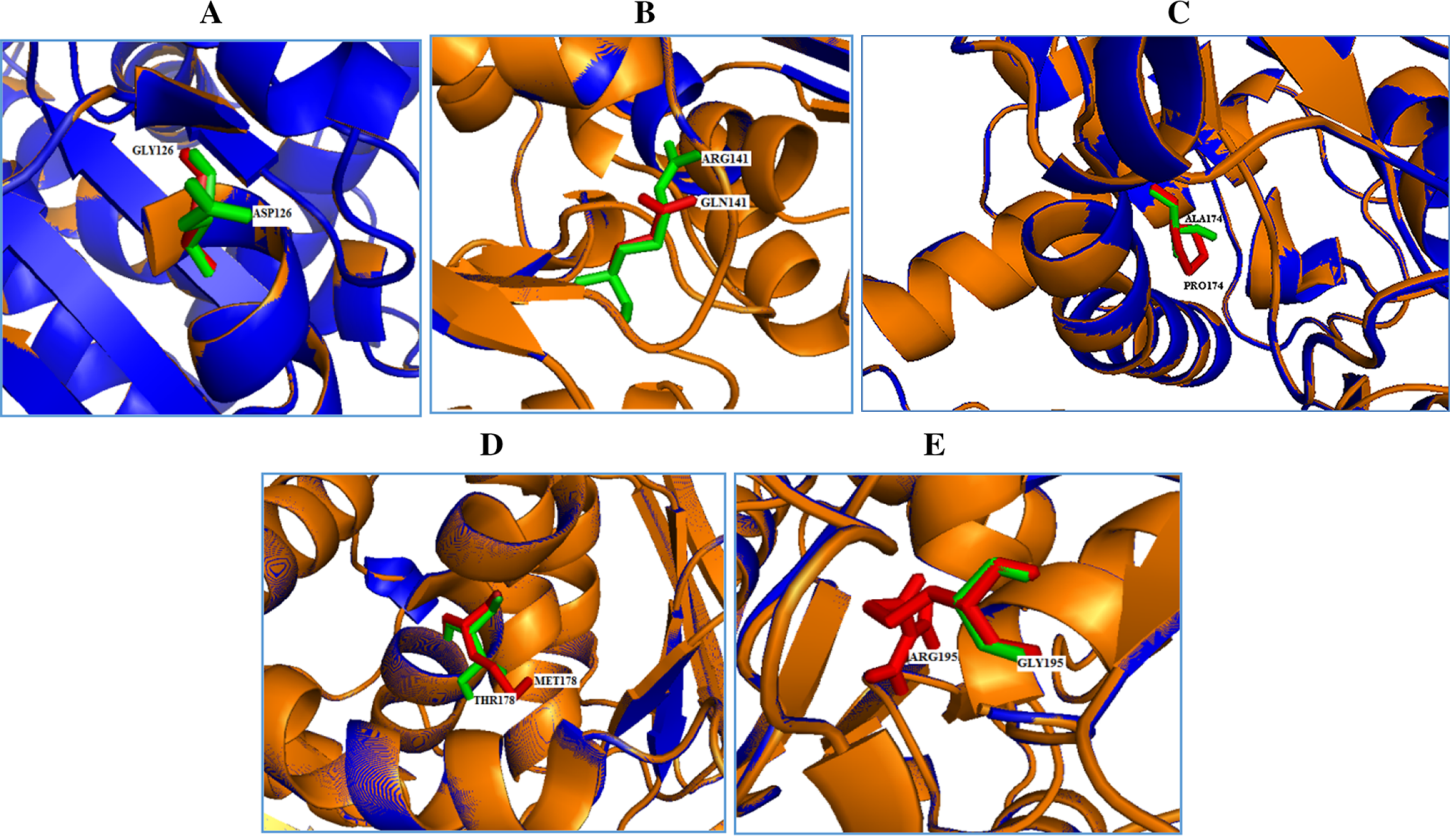
Superimposition of native and mutant modeled structures (*cartoon shape*) of OTC protein. **a** Superimposed structure of native amino acid aspartic acid (*green*) with mutant amino acid glycine (*red*) at position 126. **b** Superimposed structure of native amino acid arginine (*green*) with mutant amino acid glutamine (*red*) at position 141. **c** Superimposed structure of native amino acid alanine (*green*) with mutant amino acid proline (*red*) at position 174. **d** Superimposed structure of native amino acid threonine (*green*) with mutant amino acid methionine (*red*) at position 178. **e** Superimposed structure of native amino acid glycine (*green*) with mutant amino acid arginine (*red*) at position 195

The mutations for 1OTH at their corresponding positions were performed by SWISS-PDB viewer independently to achieve modeled structures. Then, energy minimizations were performed by NOMAD-Ref server for the native-type protein 1OTH and the mutant-type structures. The RMSD values between the native type (1OTH) and the mutant D126G is 2.01 Å, between the native type and the mutant A174P is 2.82 Å, and between the native type and the mutant G195R is 2.82 Å, respectively. The deviation between the two structures is evaluated by their RMSD values, which could affect the stability and functional activity. The RMSD values of all the mutant structures were all alike. Higher the RMSD value more will be the deviation between native- and mutant-type structures and which in turn changes their functional activity. Superimposition of native with the mutant protein D126G, R141Q, A174P, T178M and G195R of *OTC* gene is shown in (Fig. 1a–e). The total energy for the native and mutant type structures were found to be −25480.939, −24899.660, −25068.101, −24881.020, −24969.936 and −24608.215 kcal/mol respectively (Table 3).

**Table 3:** RMSD and total energy of native and mutant model of OTC gene

Native and mutant structure	RMSD (Å)	Total energy (Kcal/mol)	Stabilizing residue (Sride)
Native	0.00	–25,480.939	5
D126G	2.01	–24,899.660	3
R141Q	1.84	–25,068.101	4
A174P	2.82	–24,881.020	4
T178M	1.94	–24,969.936	3
G195R	2.82	–24,608.215	3

### Analysis of local environment changes

**Fig. 2:**
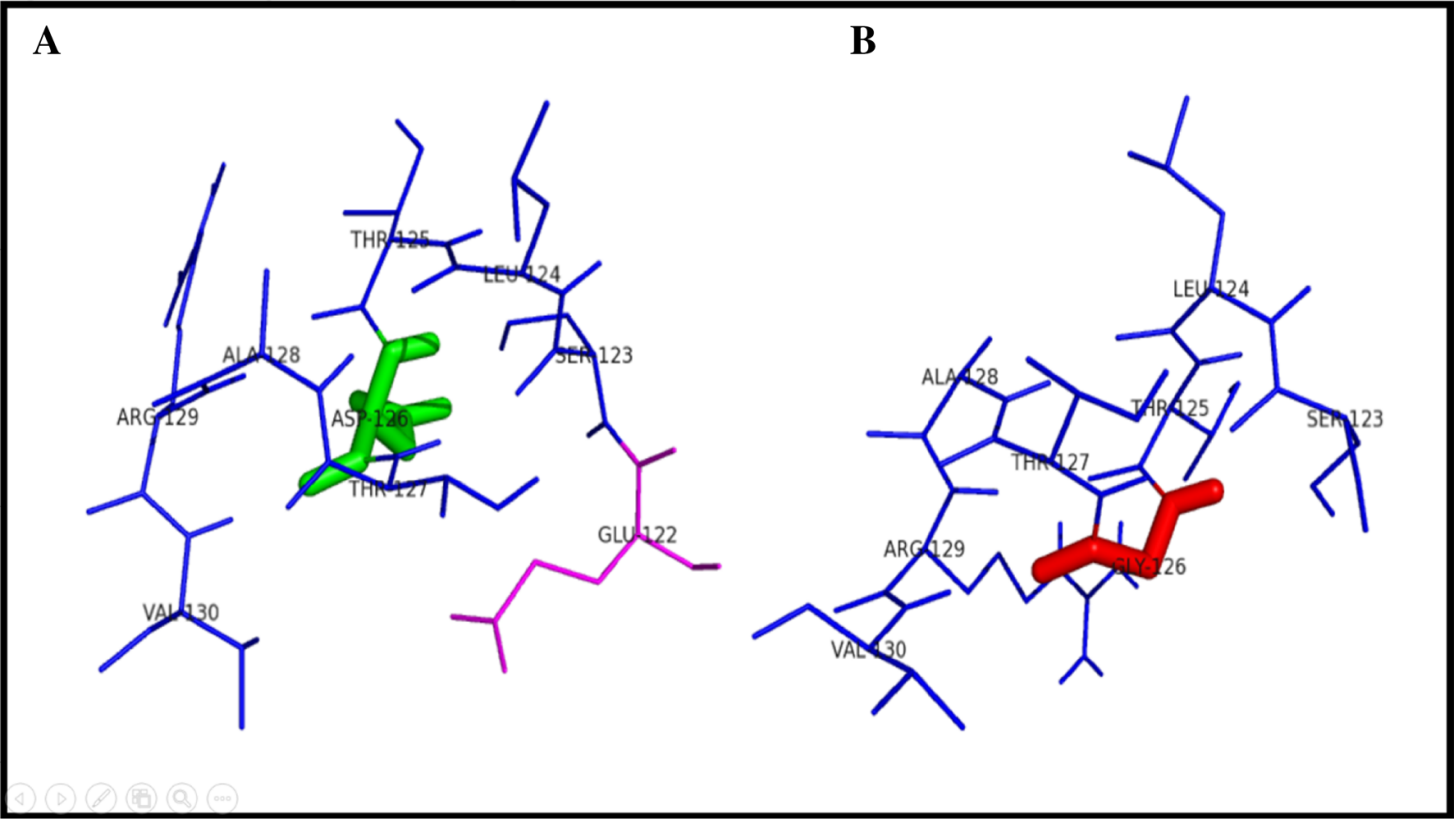
Surrounding amino acid changes in native OTC and mutant D126G structures

Within the range of 4 Å from the mutational point, surrounding amino acid changes were analyzed for native and mutant protein structures. It was observed through PyMOL (DeLano 2002). Figure 2 shows the substitution of hydrophilic residue aspartic acid to hydrophobic residue glycine at position 126, which leads to hydrophobic change at the core of the protein that could result in the destabilization of the gamma turns. The drift in hydrophilic to hydrophobic property can result in the gain of one amino acid LEU 131 in mutant structure.

**Fig. 3:**
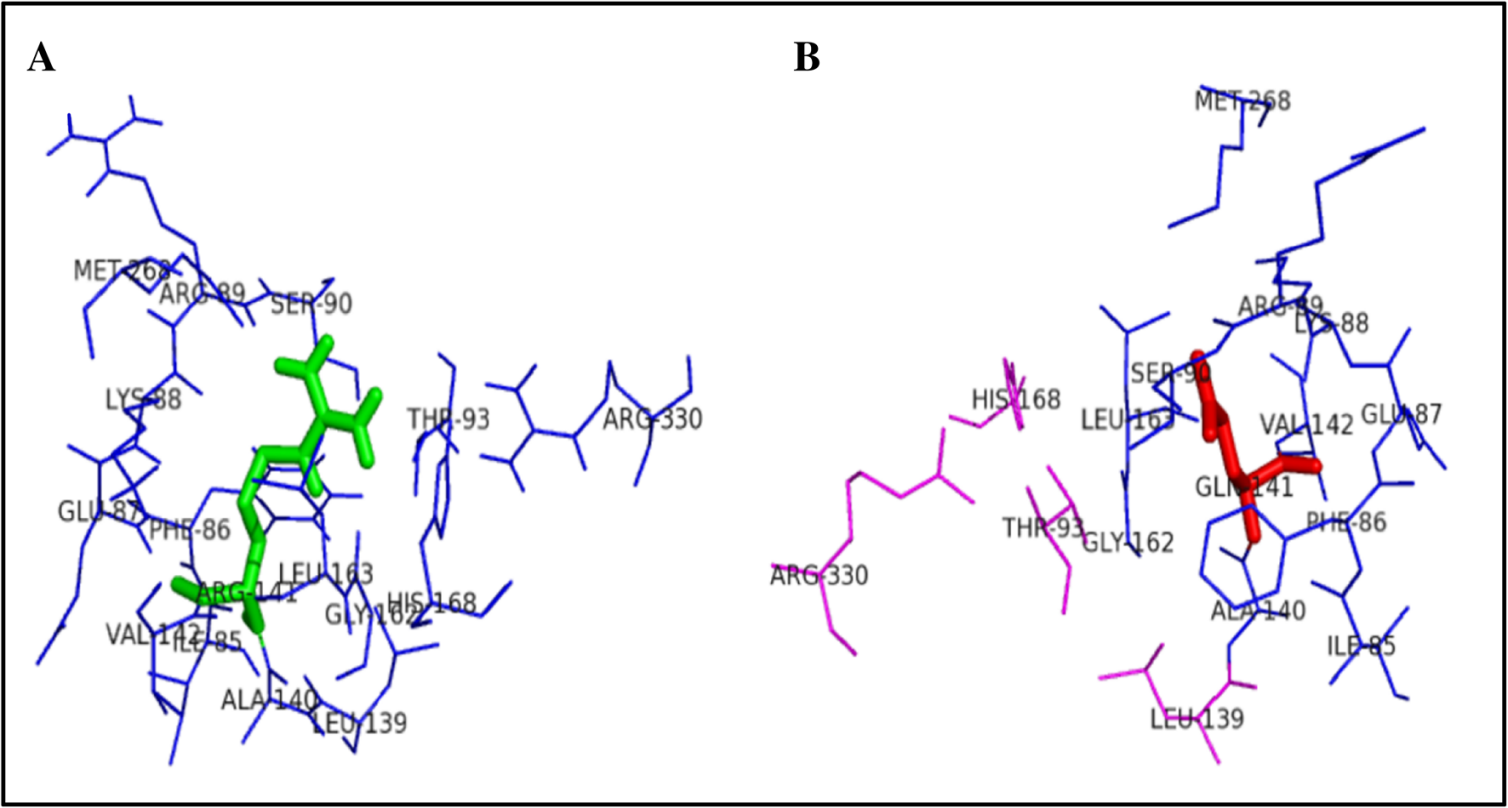
Surrounding amino acid changes in native OTC and mutant R141Q structures

**Fig. 4:**
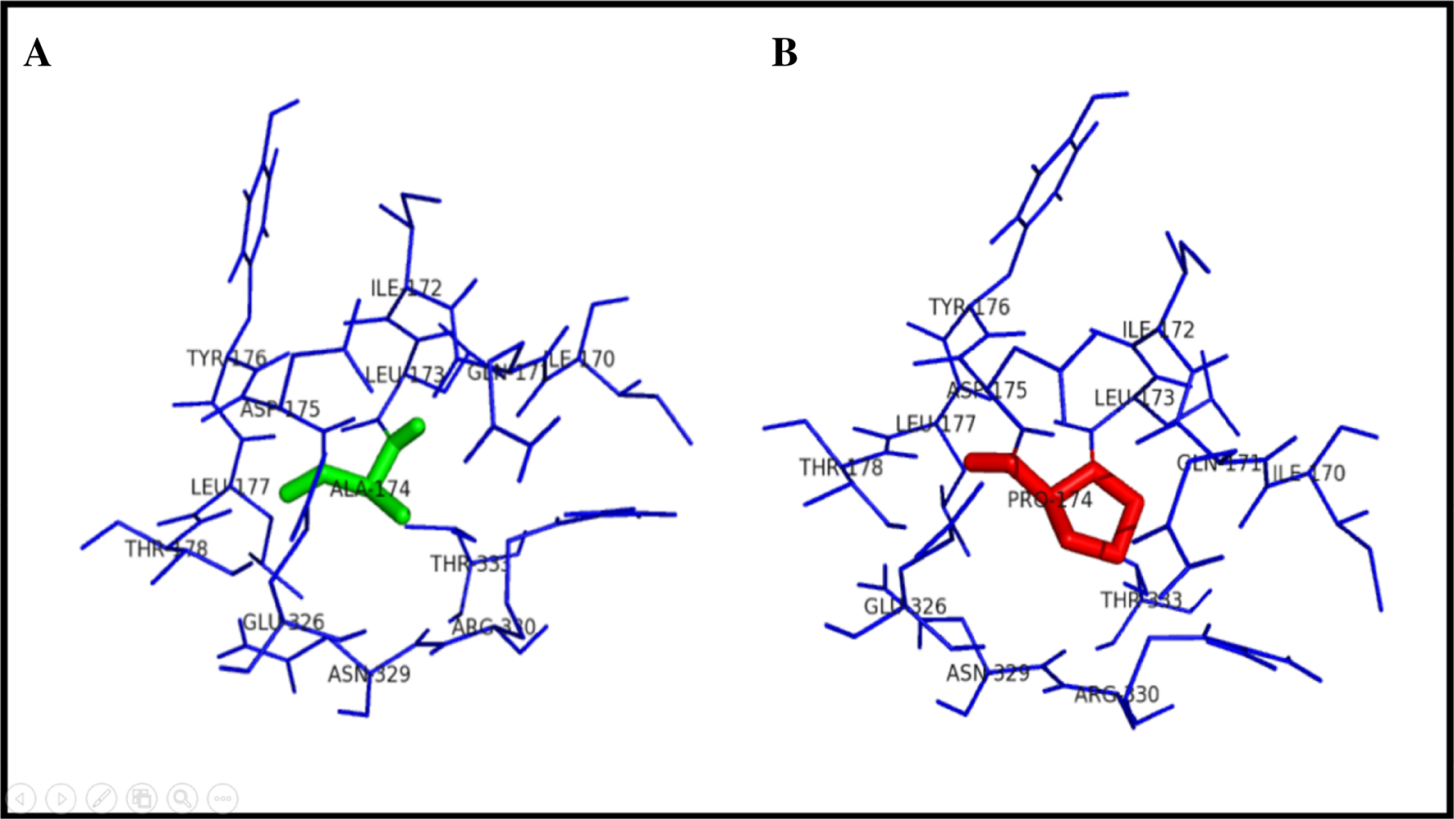
Surrounding amino acid changes in native OTC and mutant A174P structures

**Fig. 5:**
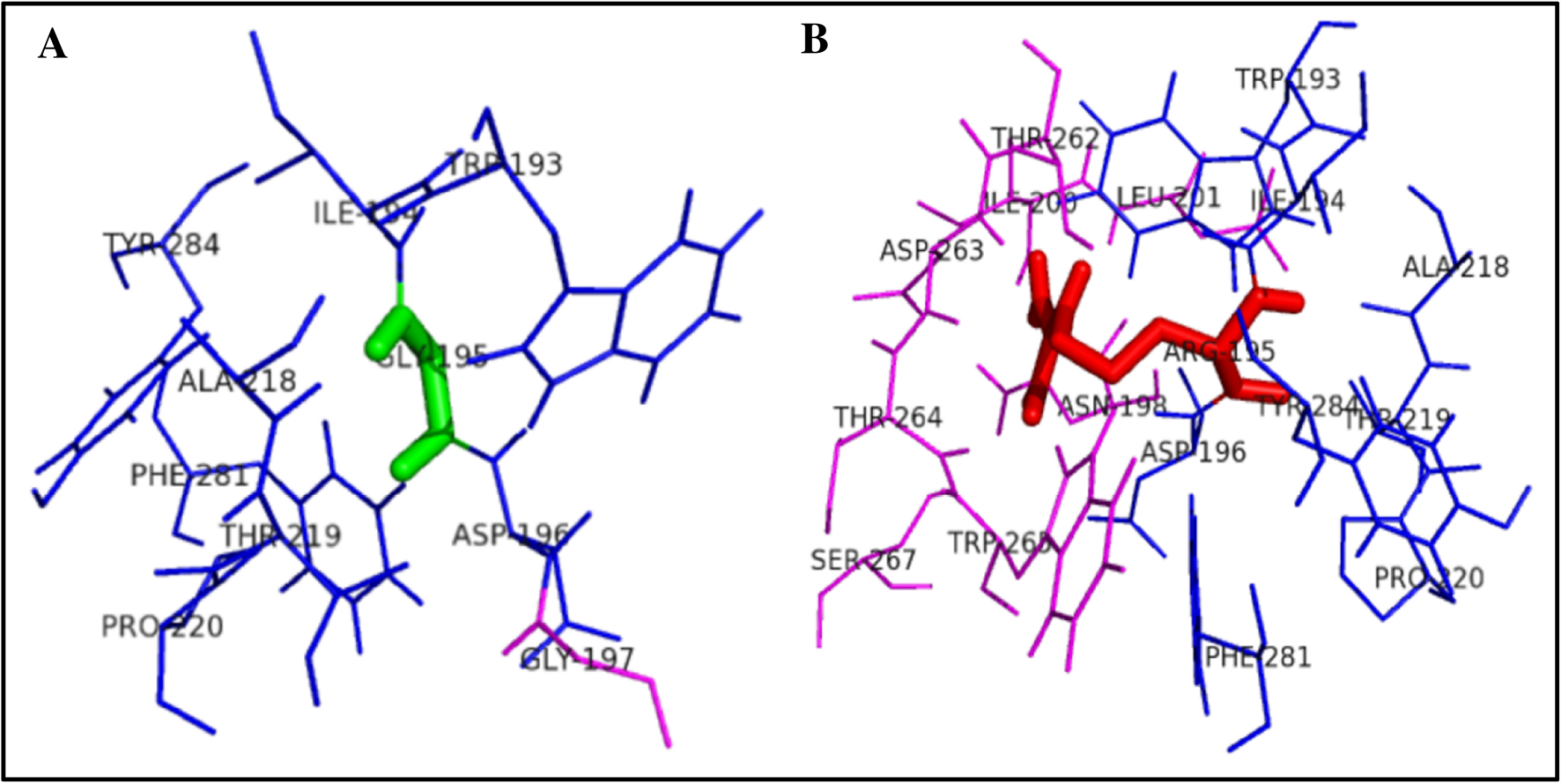
Surrounding amino acid changes in native OTC and mutant G195R structures

**Fig. 6:**
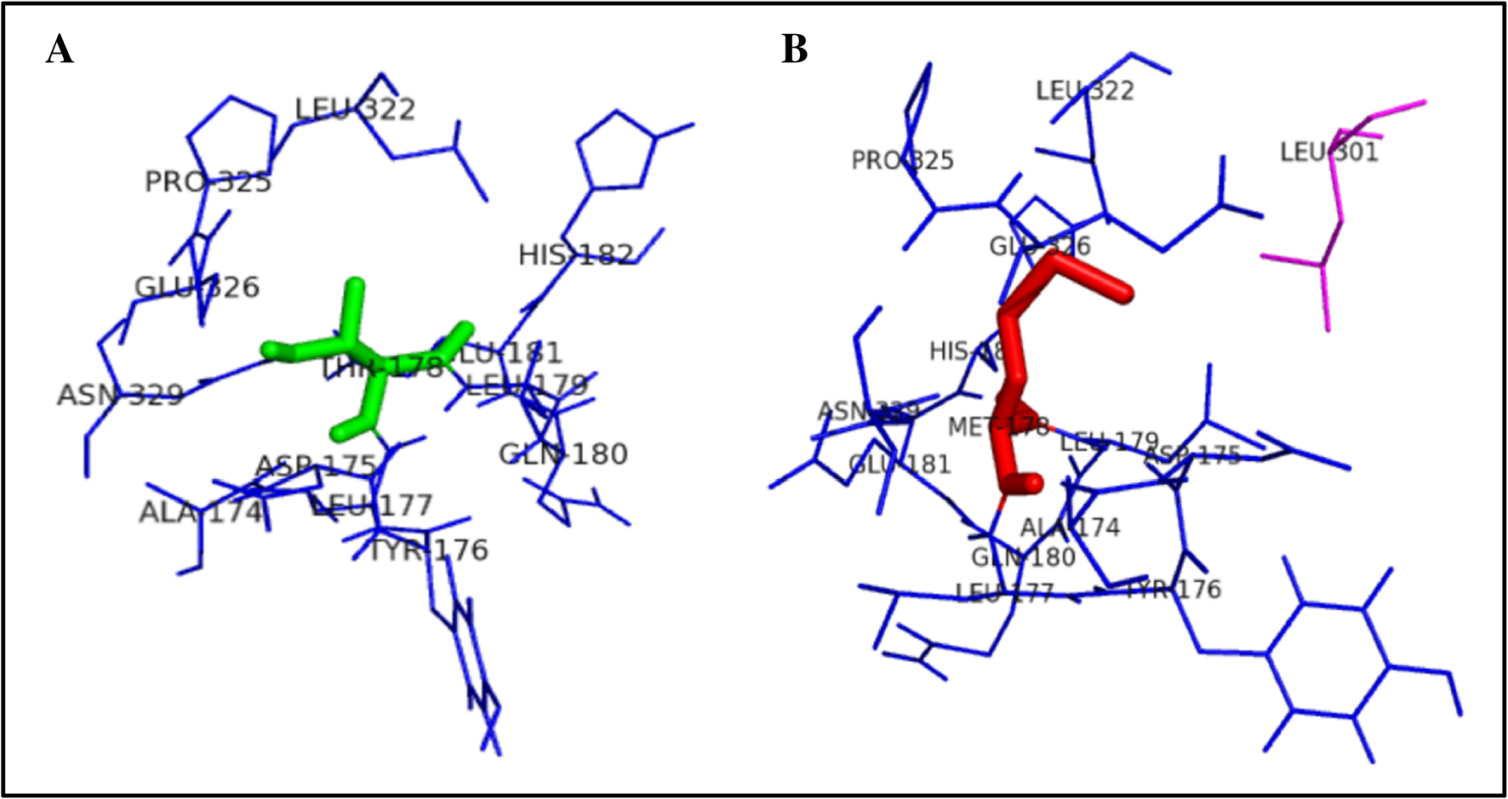
Surrounding amino acid changes in native OTC and mutant T178M structures

Figure 3 illustrates the substitution of the hydrophilic residue arginine with another hydrophilic residue glutamine at position 141, which leads to structural modification at the core region of the protein due to the size of the substituted amino acid, and that could result in affecting the strand portion. The changes in the amino acid size results in loss of four amino acids ARG330, HIS268, LEU139, and THR93 in mutant R141Q structure. Substitution of hydrophobic residue alanine with another hydrophobic residue proline and changes in the surrounding amino acids are shown in Fig. 4. Since the size of the substituted amino acid has the same size of the native residue, these changes were not affected the surrounding amino acids in A174 P-mutant structure. Figure 5 shows the substitution of non-polar hydrophobic amino acid glycine with polar hydrophilic larger amino acid arginine at position 195 of OTC protein. Substitution of small amino acid glycine with large amino acid arginine leads to gain of seven SER267, THR264, ILE200, ASP263, TRP265, ASN198, and LEU252 amino acids in the surrounding region of mutant structure. This change may affect the gamma turn of the native protein. Substitution of polar hydrophobic amino acid threonine at position 178 with non-polar hydrophobic amino acid methionine is shown in Fig. 6. This substitution leads to gain of one amino acid in the mutant structure and this change may affect the helix region of the native OTC protein.

### Secondary structural changes analysis

The number of secondary structure elements such as Beta sheets, Beta–Alpha Beta, Strands, Helices, Helix–Helix Interacs, Beta Turns, and Gamma Turns was calculated for both the native and mutant models (Table 4). It has to note that the observed numbers of secondary structural elements are equal in both native and mutant models except the Helix–Helix Interacs and Beta Turns. There was a slight decrease in the number of beta turns in mutant models D126G, R141Q, A174P, T178M, and G195R as 15, 12, 15, 12, and 15, respectively. The number of beta turn was increased by one in three mutant models R141Q, A174P, and T178M. These secondary structural element changes lead to changes in the physiochemical properties of the mutant structure (Table 5) and it may affect the protein stability and conformation.

**Table 4:** Secondary structural elements in native and mutant structure of *OTC* gene

S. no.	Variant	Sheet	Beta-alpha-beta units	Strands	Helices	Helix–Helix interacs	Beta turns	Gamma turns
1	Native	2	5	9	15	16	23	3
2	D126G	2	5	9	15	**15**	23	3
3	R141Q	2	5	9	15	**12**	**24**	3
4	A174P	2	5	9	15	**15**	**24**	3
5	T178M	2	5	9	15	**12**	**24**	3
6	G195R	2	5	9	15	**15**	23	3

Change in the secondary structure elements are highlighted in bold

**Table 5:** Changes in the physiochemical properties of native and mutant structure of OTC protein

Variant	Size	Charge	Polarity	Hydrophobicity	Disulfide bond	Gly or Pro	Modification score	Accessibility		Free energy changes	
								Wild type	Mutant	Protein stability	Reliability index
D126G	Decrease	Decrease	Decrease	Increase	Unchanged	Apparition	28	25.17 (intermediate)	11.51 (intermediate)	Increase	4
R141Q	Decrease	Decrease	Unchanged	Increase	Unchanged	Unchanged	23	4.63 (buried)	0.37 (buried)	Decrease	7
A174P	Increase	Unchanged	Unchanged	Decrease	Unchanged	Apparition	15	0.00 (buried)	0.00 (buried)	Decrease	2
T178M	Increase	Unchanged	Decrease	Increase	Unchanged	Unchanged	32	1.30 (buried)	2.71 (buried)	Decrease	1
G195R	Increase	Increase	Increase	Decrease	Unchanged	Disparition	61	0.10 (buried)	0.11 (buried)	Decrease	5

## Discussion

Last decade has witnessed the accelerated expansion of information regarding the genomic variants especially SNPs in public databases as a result of improved second generation sequencing technologies. After polymorphism information has become abundant in public databases, many groups started to develop in silico tools that would computationally calculate the properties of these polymorphisms, particularly trying to extrapolate the effect of polymorphism that has on the phenotype. If dataset on the phenotypic impact is unknown (owing to the insufficiency of clinical data or experimental) or not specified, most of the tools set out to identify whether a polymorphism is detrimental or not. Anyhow, in order for the identification, to be accurate, information had to be accumulated on the features distinguishing neutral from deleterious polymorphisms; many tools and algorithms that support large-scale analyses of SNPs (In particular nsSNPs). Various computational methods have been developed for predicting the significant missense mutations based on sequence and structural methods. With respect to the information utilized by the prediction, existing methods can be roughly grouped into three categories: ‘sequence-based’, ‘structure-based’ and ‘sequence and structure-based’, respectively. Sequence-based methods can be subcategorized into sequence homology-based and single sequence-based methods. Sequence homology-based method methods in this category calculate the probability of the substitutions based on multiple sequence alignments (Ferrer-Costa et al. 2004; Shen and Vihinen 2004). Sequence homology-based tools are derived based on the premise that essential amino acids are conserved in the protein family. Hence, changes at well-conserved positions tend to be predicted as deleterious. This probabilistic method provides information about conserved sites in evolution that are often structurally or functionally important and distinguishes between missense mutations involved in disease and those that are functionally neutral. For sequence homology-based methods, the prediction accuracy depends heavily on the availability of enough homologs in protein databases. Saunders and Baker (2002) showed that the prediction accuracy decreased significantly when fewer than 5–10 homologous sequences are available. An ideal alignment should be composed of a diverse set of orthologous sequences rather than paralogs. Structure-based methods make predictions based on structural information, especially that of amino acid side-chain conformation, over packing and residue–residue contacts (Gonzalez Diaz et al. 2005). The substitution of a wild-type residue may lead to altered chemical and physical properties, thus causing structural arrangements. The third method category combines information on the sequence features, the structural parameters and contacts to characterize the substitution. The incorporation of structural data greatly improves the quality of the multiple sequence alignment and the accuracy of prediction. This is well illustrated by PolyPhen (Ramensky et al. 2002), a multiple sequence alignment server that aligns sequences using structural information. It may outperform the single sequence-based program SIFT (Ng and Henikoff 2003) in predicting the effect of amino acid mutations. In addition to PolyPhen, diverse Web-based programs are used to predict mutation effects based on homology and three-dimensional structural models, e.g., PROMALS3D (Pei et al. 2008), 3Dcoffee (O’Sullivan et al. 2004), Expresso (Armougom et al. 2006), CLUSTALW (Thompson et al. 1994), MUSCLE (Edgar 2004), PRALINE (Simossis and Heringa 2005), SPEM (Zhou and Zhou 2005). The user only needs to provide sequences, the server runs BLAST to identify close homologues of the sequences within the PDB database.

Study of the molecular basis of diseases using experimental methods is often labor intensive, and time consuming, especially in cases where there are several missense mutations causing the disease. These studies are difficult to mount on a scale that may be required for characterizing the genetic variants and at times these results might not always reflect the in vivo genotype function in humans. In contrast, precise and useful information about the effects of mutations on protein structure and function can be readily obtained by in silico methods. Our study gains significance by predicting the possible deleterious SNPs in OTC gene, so that the number of SNPs screened for association with diseases can be reduced to those that are most likely to alter gene function. All the above methods defined here follow a similar technique in which each SNP is first labeled with the properties related to damage it may cause on protein structure and function. The resulting feature vector is then used to determine whether a single residue substitution has any effect on protein function or not. Considering SNPs based on the amino acid properties are generally reflected to be an important phenomenon in defining the protein folding, stability, and its function. The results from this paper signify the impact of mutations in *OTC* gene in causing OTCD. Further, studies possibly will help in uncluttered nature of OTCD. It is hoped that the results obtained from this study would pave the way by providing useful information to the researchers, and can play an important role in bridging the gap between biologists and bioinformaticians.
